# Tuberculosis notification rate decreases faster in residents of native origin than in residents of foreign origin in the EU/EEA, 2010 to 2015

**DOI:** 10.2807/1560-7917.ES.2017.22.12.30486

**Published:** 2017-03-23

**Authors:** V Hollo, J Beauté, C Ködmön, MJ van der Werf

**Affiliations:** 1 European Centre for Disease Prevention and Control (ECDC), Stockholm, Sweden

**Keywords:** Tuberculosis, EU/EEA Member States, surveillance, migration, migrant, foreign origin

## Abstract

To estimate trends in tuberculosis (TB) notification rates by geographical origin, we retrieved surveillance data from 2010 to 2015 for 29 European Union and European Economic Area countries. The TB notification rate decreased at an annual rate of 5.3%. The decrease in notification rate was higher in native residents (7.0%) than in those of foreign origin (3.7%). Targeted screening and facilitated access to care and treatment could help prevent and control TB in migrants.

The tuberculosis (TB) notification rate in the European Union (EU) and European Economic Area (EEA) has been decreasing consistently since 2002 at an annual rate of around 5% [1]. In 2015, the EU/EEA notification rate was 11.7 per 100,000 population, close to the ‘End TB 2035’ target of less than 10 cases per 100,000 set by the World Health Organization (WHO) [2]. This encouraging figure masks important disparities both across and within countries. In 2015, rates were already below 10 per 100,000 in 22 countries but still above 50 per 100,000 in Lithuania and Romania [1]. Studies have also identified vulnerable groups for TB in low-incidence countries, such as prison inmates, people living with HIV, or migrants [3]. Here, we report TB notification rate trends for both native and foreign residents of the EU/EEA and assess progress towards TB elimination by predicting TB notification rates to 2025.

## Tuberculosis surveillance in the EU/EEA

The surveillance of TB in Europe is carried out by the European Tuberculosis Surveillance Network under the joint coordination of the European Centre for Disease Prevention and Control (ECDC) and the WHO. Each year, 30 EU/EEA countries upload all TB cases meeting the EU case definition [4] to a database hosted by ECDC (the European Surveillance System, TESSy). Information collected includes main epidemiological (time, place, sex, age, patient origin) and case management variables such as laboratory results or treatment outcome. A more detailed description of data collection methods is available elsewhere [1]. In most EU/EEA countries, a TB case of foreign origin is a case with a country of birth different from the reporting country. For Austria, Belgium, Greece, Hungary and Poland, a TB case of foreign origin is a case with citizenship different from the reporting country. For the purpose of this analysis, we included all TB cases reported for the period from 2010 to 2015. Data for Croatia were excluded because case-based data were only available from 2012 onwards.

## Population data and analysis

We obtained population denominator data by origin from the Statistical Office of the European Union (Eurostat) [5]. We used population by country of birth for most countries and population by citizenship for Austria, Belgium, Greece, Hungary and Poland. Where population data were missing (Bulgaria in 2010 and Norway in 2015), we used the data of the year after for Bulgaria and the year before for Norway. We estimated annual rates of change by origin and their 95% confidence intervals (CI) using a log-linear regression of notification rates over the period 2010 to 2015. Assuming constant rates of decrease, we estimated notification rates by origin until 2025. We did not forecast until 2035 (target year of the End TB strategy) because only six years of denominator data were available.

## Trends

Over the period from 2010 to 2015, 29 countries reported 404,551 TB cases, of which 394,110 (97.4%) had information on origin. Of these 394,110 cases, 283,426 (71.9%) were born in or citizens of the reporting country and 110,684 (28.1%) were of foreign origin (Table).

**Table:** Number and rate of tuberculosis cases per 100,000 population and population by origin, EU/EEA, 2010−2015 (n = 404,551)^a^

Year	Native			Foreign origin			Unknown origin^b^	Total		
	Cases	Population (million)	Rate	Cases	Population (million)	Rate	Cases	Cases	Population (million)	Rate
2010	54,956	456.9	12.0	19,242	47.5	40.5	1,376	75,574	504.4	15.0
2011	52,753	457.3	11.5	19,504	47.1	41.4	1,045	73,302	504.4	14.5
2012	49,498	457.4	10.8	19,038	48.2	39.5	994	69,530	505.6	13.8
2013	44,877	456.7	9.8	17,742	48.9	36.3	2,549	65,168	505.5	12.9
2014	41,870	457.8	9.1	17,319	49.5	35.0	2,079	61,268	507.3	12.1
2015	39,472	458.0	8.6	17,839	50.9	35.1	2,398	59,709	508.9	11.7

EU/EEA: European Union/European Economic Area.

^a^ Croatia excluded.

^b^ Without denominator, rates were not calculated for cases of unknown origin.

The proportion of cases of foreign origin continuously increased from 25.9% in 2010 to 31.1% in 2015. Over the same period, the proportion of EU residents of foreign origin remained stable at 9.4% in 2010 and 10.0% in 2015. Overall, the TB notification rate decreased at an annual rate of 5.3% (95% CI: 4.4–6.1) over the study period. This decrease was more pronounced in native residents (7.0%, 95% CI: 6.0–8.0) than in cases of foreign origin (3.7%, 95% CI: 1.7–5.8). The rate ratio of TB cases of foreign origin over native residents increased from 3.4 in 2010 to 4.1 in 2015. Assuming that similar decreases in notification rates would be observed in the following years, the overall TB notification rate would cross the 10 per 100,000 threshold by 2018 (Figure).

**Figure 1:**
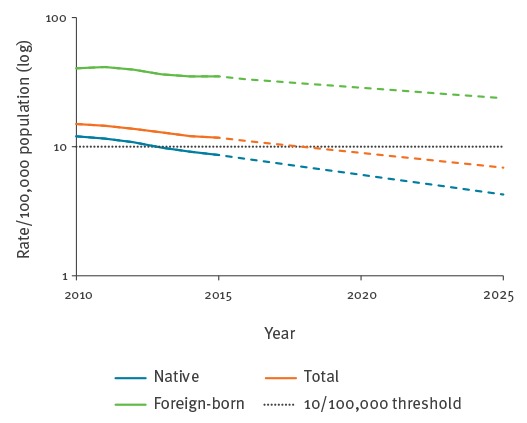
Notification rate of tuberculosis cases per 100,000 population, by year and origin, EU/EEA, 2010–2015, and prediction for 2016−2025

EU/EEA: European Union/European Economic Area.

Solid lines: rates calculated from reported cases; dotted lines: estimated prediction.

By 2025, the estimated notification rate in native residents would be at 4.3 per 100,000, approaching the pre-elimination target of less than 1 case per 100,000 [3]. However, the notification rate in cases of foreign origin would still be higher than 20 cases per 100,000.

## Discussion

The TB notification rate is decreasing in the EU/EEA, but the pace differs depending on cases’ geographical origin. Residents of foreign origin have a three- to fourfold higher notification rate compared with natives. This was observed in most countries except Bulgaria, Hungary, Latvia, Lithuania, Poland and Romania, where higher TB notification rates were reported in natives. Studies have suggested that TB rates in migrants are strongly associated with the incidence in their country of origin [6,7]. It is therefore not surprising to observe high rates of TB in residents of foreign origin in some EU/EEA countries because a considerable proportion of them originate from high-TB-incidence countries [8]. Since 2000, TB incidence has also been decreasing globally but at a slower rate than in EU/EEA countries [2]. Thus, TB cases of foreign origin are and will remain a challenge for TB elimination, especially in low-incidence countries where they account for a substantial proportion of TB cases [9].

The main reason explaining the higher TB burden in residents of foreign origin in high-income countries is thought to be reactivation of remotely acquired latent tuberculosis infection [10]. This does not exclude other possible explanations such as travel-associated infection when visiting friends or relatives in the country of origin [11] or infection in the receiving country where migrants may face poor living conditions. The latter two reasons could also partly explain why also second-generation migrants may be at higher risk for TB infection compared with native residents [12].

The main limitation of this analysis is that we classified all cases with a birthplace different from the reporting country as cases of foreign origin regardless of their time of arrival or the duration of their stay in the receiving countries. Also, we were not able to distinguish between migrants from low- and high-TB incidence countries. Characteristics of migrants and travellers are of increasing complexity which is challenging to capture through binary variables. Global travel and migration patterns have changed and intra-regional migration has increased [8]. Migrants may have stayed in other countries on their journey to the receiving country and been exposed to TB in other places than their country of origin. Estimates at EU/EEA level may mask important disparities across countries in which patterns of migration differ.

To address the challenge of TB among migrants in low-incidence countries, targeted prevention and control strategies should be implemented taking into account the origin of migrants but also their demographic characteristics. As most cases of foreign origin are likely to have been infected in their country of origin, preventive strategies in the host countries may have limited impact on the overall notification rate. A recent review suggested that targeted pre-arrival screening for active TB and post-arrival screening for latent TB infection in migrants would be the most efficient strategy [10]. Strategies reaching migrants arriving through irregular channels should also be explored.

## Conclusion

The TB notification rate in individuals of foreign origin reported by EU/EEA countries is higher, and decreasing at a slower pace, than in native residents. This will be one of the main challenges for EU/EEA countries when trying to reach the TB elimination target in the coming years, especially in countries where individuals of foreign origin account for a large proportion of TB cases. Targeted screening and facilitated access to care and treatment could help tackle this issue.
